# Plasma osteopontin versus intima media thickness of the common carotid arteries in well-characterised patients with systemic lupus erythematosus

**DOI:** 10.1177/09612033211013898

**Published:** 2021-05-06

**Authors:** Lina Wirestam, Muna Saleh, Christina Svensson, Michele Compagno, Helene Zachrisson, Jonas Wetterö, Christopher Sjöwall

**Affiliations:** 1 Division of Inflammation and Infection/Rheumatology, Department of Biomedical and Clinical Sciences, Linköping University, Linköping, Sweden; 2 Department of Clinical Physiology, University Hospital and Department of Health, Medicine and Caring Sciences, Linköping University, Linköping, Sweden; 3 Department of Clinical Sciences Lund, Rheumatology, Lund University, Lund, Sweden

**Keywords:** Osteopontin, carotid intima-media thickness, atherosclerosis, systemic lupus erythematosus, biomarker

## Abstract

**Objective:**

The progress of accelerated atherosclerosis in systemic lupus erythematosus (SLE) is incompletely understood. Circulating osteopontin (OPN) is increased in autoimmune conditions, e.g. SLE, and its serum concentration was recently reported to associate with subclinical atherosclerosis in SLE, as measured by carotid intima-media thickness. The aim of this study was to investigate whether OPN may be used as a surrogate biomarker of subclinical atherosclerosis in SLE patients with different disease phenotypes.

**Methods:**

We recruited 60 well-characterised SLE cases and 60 age- and sex-matched healthy controls. The SLE cases were divided into three different disease phenotypes: SLE with antiphospholipid syndrome (APS), lupus nephritis, and isolated skin and joint involvement. Plasma OPN was detected by ELISA (Quantikine®, R&D Systems). Common carotid arteries intima media thickness was compared between the studied groups in relation to OPN levels and risk factors for vascular changes. Intima media thickness of common carotid arteries was measured by using a sensitive ultrasound technique (LOGIQ™ E9 ultrasound, GE Healthcare).

**Results:**

OPN levels were significantly higher among the entire SLE group (*n* = 60) compared to the healthy controls (*P* = 0.03). SLE cases with concomitant APS (*n* = 20) showed higher OPN levels than the controls (*P* = 0.004), whereas none of the other two subgroups differed significantly from the healthy controls. OPN and intima media thickness were correlated to several traditional risk factors of atherosclerosis, as well as to SLE-related factors. Yet, no significant correlation was observed between OPN levels and ultrasound findings of the common carotid arteries.

**Conclusions:**

In line with previous studies, we observed increased OPN levels among SLE patients as compared to matched controls. However, the OPN concentrations did not correlate with intima media thickness of the common carotid arteries. Based on our findings, the use of OPN as a surrogate biomarker of subclinical atherosclerosis in SLE subjects, regardless of clinical phenotypes, cannot be recommended.

## Introduction

Systemic lupus erythematosus (SLE) is an autoimmune inflammatory disease that usually affects young females and may impair several organ systems, with an increased risk of cerebro- and cardiovascular disease (CVD).^1–3^ Atherosclerosis is an inflammatory process with immune cell activation, leading to plaque formation and risk of subsequent rupture.^4^ The systemic inflammation occurring in patients with SLE is thought to accelerate atherosclerosis,^1,5,6^ but additional mechanisms are probably involved. The autoimmune vascular injury may facilitate the atherosclerotic plaque formation. Furthermore, in SLE patients with or without concomitant antiphospholipid syndrome (APS), the excessive oxidative stress, the apoptotic cell death and the defective clearance of apoptotic materials contribute to tissue damage, and dyslipidaemia can further accelerate atherogenesis.^7–9^ The pivotal role of the type I interferon (IFN) system may also promote atherosclerosis, by a pro-inflammatory action on the endothelium and by stimulating macrophage recruitment to atherosclerotic lesions.^10,11^

Traditional risk factors for atherosclerosis, e.g. diabetes, hyperlipidaemia, hypertension, family history CVD, obesity, and tobacco smoking, along with the common need of glucocorticoid treatment, may even more increase the risk of CVD in subjects affected by SLE.^12–14^ In addition, vasculitis primarily affecting small vessels is not uncommon; medium and large sized vessels are less often engaged by vasculitis in SLE.^15^

The extracellular matrix protein osteopontin (OPN) is a mediator of systemic inflammation and has multiple biological functions.^16^ Local production and elevated circulating levels of OPN have been observed in several autoimmune diseases, including SLE.^17,18^ Overexpression of OPN in lupus-prone mice induces B-cell activation and subsequent production of anti-double-stranded (ds) DNA antibodies, a distinctive laboratory finding in subjects with SLE. Intracellular OPN has been implicated in many cellular processes and its expression is required for Toll-like receptor 9-dependent production of IFN-α.^19^ Two recent studies by Carbone *et al.* suggested OPN as a potential predictor of poor outcome in patients with severe carotid atherosclerosis^20^ and as a valuable biomarker in SLE, showing a strong association with subclinical atherosclerosis, measured as carotid intima-media thickness (IMT).^21^ In contrast, OPN plasma levels (pOPN) and early vascular markers of atherosclerosis in asymptomatic young Scandinavian adults were poorly correlated.^22^

The high-frequency ultrasound (HFUS) facilitates the mapping of the vascular damage and gives the clinician the opportunity to distinguish atherosclerosis from inflammation, in individuals with SLE and other inflammatory conditions. Progression of IMT, as well as the presence of carotid plaques, has been associated with traditional cardiovascular risk factors, besides SLE per se.^23^ We have recently shown that an extended HFUS protocol focused on multiple arterial areas may provide the clinician with additional information on the vessel wall appearance, in SLE subjects.^24^ We recorded that increased IMT (≥0.9 mm) observed in SLE predominantly showed appearance of a medium echogenic, homogenous wall thickening that can be found in inflammatory vascular disease. Concerning early wall changes in SLE, our results indicated other potential mechanisms apart from atherosclerosis.^24^

Herein, we aimed to investigate the reliability of pOPN as a surrogate biomarker of atherosclerosis in the common carotid arteries (CCA) of subjects with different SLE phenotypes i.e. in patients with either APS, or nephritis (LN), or isolated skin/joint involvement, as well as in matched control subjects.

## Materials and methods

### Study population

Patients in this cross-sectional study were recruited from the observational research program KLURING (a Swedish acronym for *Clinical LUpus Register in Northeastern Gothia*), in which prevalent and incident SLE cases continuously have been included and longitudinally followed since 2008 at the Rheumatology Unit, Linköping University Hospital.^25^ Sixty patients (52 women, 8 men; median age 43.0 and mean 42.9 years; range 23–63 years) and 60 age- and sex-matched healthy controls (see below) were recruited. All patients were diagnosed with SLE and fulfilled the 1982 American College of Rheumatology (ACR) and/or the 2012 Systemic Lupus International Collaborating Clinics (SLICC) classification criteria as detailed in Table 1.^26^ In each patient, the acquired organ damage was assessed by the SLICC/ACR damage index (SDI)^27^ and the disease activity by the SLE disease activity index 2000 (SLEDAI-2K).^28^

**Table 1.** Detailed characteristics of the included patients and healthy controls presented as mean ± SD or *n* (%).

	All SLE(*n* = 60)	Controls (*n* = 60)	LN (*n* = 20)	APS (*n* = 20)	Skin and joint (*n* = 20)
Variables	Mean ± SD	Mean ± SD	Mean ± SD	Mean ± SD	Mean ± SD
Background variables					
Age at examination (years)	43.2 ± 11.3	43.0 ± 11.4	41.6 ± 10.4	45.2 ± 12.2	42.9 ± 11.7
Female gender, *n* (%)	52 (87)	52 (87)	18 (90)	15 (75)	19 (95)
Duration of SLE (years)	12.0 ± 9.4	NA	10.7 ± 8.1	15.6 ± 12.2	9.6 ± 6.3
SDI score	0.8 ± 1.1	NA	0.6 ± 0.9	1.5 ± 1.4	0.4 ± 0.5
SLEDAI-2K	2.0 ± 2.1	NA	1.6 ± 2.1	2.1 ± 2.4	2.2 ± 1.7
Traditional risk factors and laboratory data					
Body mass index (BMI) (kg/m²)	26.0 ± 4.2^a^	24.0 ± 3.3	26.5 ± 3.4^b^	25.6 ± 4.0	25.8 ± 5.1
Ever smoker (former or current), *n* (%)	14 (23)	0	4 (20)	3 (15)	7 (35)
Systolic blood pressure (mm Hg)	115 ± 26	112 ± 18	117 ± 17	113 ± 32	116 ± 29
Diastolic blood pressure (mm Hg)	73 ± 11^b^	68 ± 8	74 ± 12	73 ± 10	72 ± 9
Diabetes mellitus, *n* (%)	1 (2)	0	0	1 (5)	0
Raynaud, *n* (%)	16 (27)	9 (15)	4 (20)	5 (25)	7 (35)
eGFR (mL/min/1,73m²)	84 ± 16	NA	85 ± 14	79 ± 18	87 ± 13
Total cholesterol (mmol/L)	4.7 ± 1.0	4.9 ± 1.1	4.5 ± 1.0	4.7 ± 0.8	4.9 ± 1.1
High-density lipoprotein (HDL) (mmol/L)	1.6 ± 0.5	1.7 ± 0.4	1.5 ± 0.4	1.6 ± 0.5	1.6± 0.4
Low-density lipoprotein (LDL) (mmol/L)	2.6 ± 0.8	2.6 ± 0.9	2.5 ± 0.9	2.5 ± 0.7	2.9 ± 0.9
Triglycerides (TG) (mmol/L)	1.1 ± 0.7	1.2 ± 0.6	1.2 ± 0.6	1.3 ± 1.0	0.9 ± 0.4
hsCRP (mg/L)	2.2 ± 2.8	2.0 ± 3.7	1.4 ± 1.3	2.7 ± 3.4	2.5 ± 3.2
Medical treatment, ongoing					
Antimalarial agents, n (%)	54 (90)	0	20 (100)	16 (80)	18 (90)
Glucocorticoid therapy *n* (%)	31 (52)	0	12 (60)	9 (45)	10 (50)
*Mean daily Prednisolone dose (mg)*	4.5	0	5.4	3.8	4.2
Warfarin therapy, *n* (%)	11 (18)	0	1 (5)	10 (50)	0
Antiplatelet therapy, *n* (%)	11 (18)	0	5 (25)	6 (30)	0
Statin therapy *n* (%)	5 (8)	0	2 (10)	3 (15)	0
DMARD therapy, *n* (%)	27 (45)	0	11 (55)	9 (45)	7 (35)
Mycophenolate mofetil, *n* (%)	16 (27)	0	11 (55)	4 (20)	1 (5)
Methotrexate, *n* (%)	5 (8)	0	0	1 (5)	4 (20)
Azathioprine, *n* (%)	3 (5)	0	0	2 (10)	1 (5)
Sirolimus, *n* (%)	2 (3)	0	0	1 (5)	1 (5)
Dehydroepiandrosterone, *n* (%)	1 (2)	0	1 (2)	0	0
Biologics, *n* (%)	4 (7)	0	3 (15)	1 (5)	0
Bortezomib, *n* (%)	1 (2)	0	1 (5)	0	0
Rituximab, *n* (%)	1 (2)	0	1 (5)	0	0
Belimumab, *n* (%)	2 (3)	0	1 (5)	1 (5)	0

APS: antiphospholipid syndrome; hsCRP: high-sensitivity C-reactive protein; DMARDs: disease modifying anti-rheumatic drugs; eGFR: estimated glomerular filtration rate; LN: lupus nephritis; N/A: not applicable or available; SDI: SLICC/ACR damage index; SLE: systemic lupus erythematosus.

^b^*p* < 0.05.

^a^*p* < 0.01.

The selected patients were further divided in three phenotypic subgroups, based on the main clinical manifestations. The subgroups were matched between each other 1:1:1 according to sex and age; 20 cases meeting the renal disorder ACR criterion for LN^29^ in the absence of APS, 20 cases meeting APS criteria^30^ in the absence of LN, and 20 cases with skin and joint involvement in the absence of LN and APS. Immediately next to the HFUS examination, peripheral venous blood was drawn from each individual, and plasma was prepared and stored at −70°C until analysed.

Sixty healthy Caucasian, age- and sex-matched (1:1 to the 60 SLE cases), non-medicated controls (52 women and 8 men; median age 43.0 and mean 42.9 years; range 23–63 years) were examined with HFUS and blood tests, using the same protocol as for the patients. None of them had clinical signs of inflammatory or atherosclerotic disease.

### OPN immunoassay

A serum- and plasma-validated ELISA kit (Quantikine, R&D Systems, Minnesota, USA) was used to analyse pOPN in SLE and control plasma. The samples were randomly applied on the ELISA plates and all assays were performed by the same person (LW) in Linköping, according to the manufacturers’ instructions, as previously described.^31^ A correlation coefficient of 0.77 was achieved by measuring OPN in individuals from whom both serum and plasma had been collected simultaneously (Suppl. Figure 1).

**Figure 1.**
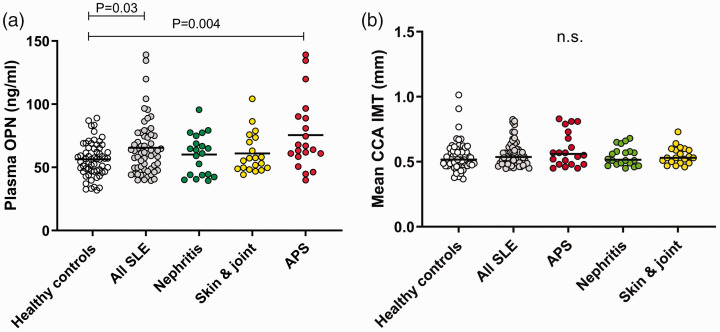
(a) Plasma osteopontin (OPN) concentrations in healthy controls (*n* = 60) and the entire group of SLE patients (*n* = 60), as well as for the three SLE phenotypes of which each include 20 individuals. (b) CCA IMT of healthy controls (*n* = 60) and the entire group of SLE patients (*n* = 60), as well as for the three SLE phenotypes of which each include 20 individuals. n.s. = not significant.

### High-frequency ultrasound

For the HFUS measurements, a LOGIQ™ E9 XDclear 2.0 (General Electric Medical Systems Ultrasound, Wauwatosa, WI, USA) ultrasound system was used, with linear transducers L2-9 MHz. The scan was performed in both transverse and longitudinal planes with the patient lying in the supine position with neck extension. The image was carefully optimized with focus on the arterial vessel wall, a preinstalled software with high frequency, medium frame rate and medium dynamic range was used. IMT was measured in CCA. Both sides were investigated. The wall thickness was measured in the longitudinal plane with a 10 mm measurement box placed over the common carotid artery far wall, near (10 mm) the carotid bifurcation. A mean value of all measured far wall points in the box is presented. Two repeated measurements were performed on each side by the same examiner (C.Sv.). Mean CCA IMT bilaterally were used.

### Background variables

We obtained data from patients and controls regarding traditional risk factors potentially contributing to atherosclerosis, such as height and weight presented as Body Mass Index (BMI). Variables concerning age, sex, smoking habits, diabetes, presence of Raynaud’s phenomenon and ongoing pharmacotherapy (antimalarial agents, glucocorticoids, warfarin, antiplatelet therapy, statins and disease modifying anti-rheumatic drugs (DMARDs)) were also collected. Blood pressure was determined with oscillometric technique (Dinamap PRO 200 Monitor, Critikon, Tampa, FL, USA).

### Laboratory measurements

Blood samples were collected after 12-h overnight fasting at the same day as HFUS examination. Standard measurements of total cholesterol, triglycerides, high-density lipoprotein (HDL), low-density lipoprotein (LDL), plasma creatinine, and C-reactive protein by high sensitive technique (hsCRP) were performed at the Clinical Chemistry laboratory, at Linköping University Hospital, Sweden. The 4-variable Modification of Diet in Renal Disease Study equation based on plasma creatinine was used to estimate the glomerular filtration rate (eGFR).^32^

Presence of anti-dsDNA antibodies (using addressable laser bead immunoassay FIDIS™ Connective profile, Solonium software version 1.7.1.0, Theradiag, Croissy-Beaubourg, France) and plasma levels of complement proteins (C3 and C4) were assessed as serological markers of disease activity.^33^

### Statistics

A univariate linear regression model was used to evaluate correlations and predictive effects of the investigated risk factors for atherosclerosis in relation to pOPN and CCA IMT. The factors with *P* values ≤0.05 in the univariate analysis were included in a multivariable regression analysis. Correlations between pOPN and CCA IMT were examined by linear regression analysis. Statistical analyses were performed using SPSS Statistics V.26 (IBM, Armonk, New York, USA) or GraphPad Prism, V.8 (GraphPad Software, La Jolla, CA, USA).

### Ethics considerations

Oral and written informed consent was obtained from all patients and healthy controls. The study protocol was approved by the Regional Ethics Review Board in Linköping (Decision No. M75-08).

## Results

### Descriptive data

A detailed descriptive statistical analysis including demographics, clinical features and medications of the included groups and subgroups is presented in Table 1.

### pOPN in different SLE phenotypes versus controls

pOPN levels were significantly higher among patients with SLE (median 61.5 ng/ml) compared to the healthy controls (median 56.6 ng/ml, *P* = 0.03; Figure 1(a)). No statistically significant differences were observed between the SLE phenotype subgroups. By comparison of each subgroup with the controls, significantly higher pOPN levels were detected among individuals with APS (*P* = 0.004; Figure 1(a)).

### Correlation of pOPN in all patients

For the entire group of patients (*n* = 60), pOPN was significantly correlated with several variables, including SLE disease duration, SDI, hsCRP, occurrence of Raynaud’s phenomenon and ongoing warfarin therapy (Table 2). No significant associations were found between pOPN levels and any of the studied variables among the controls.

**Table 2.** Plasma osteopontin levels related to background variables, traditional risk factors, laboratory tests and pharmacotherapy in the univariate regression model of SLE cases with subgroups compared to healthy controls.

	All SLE(*n* = 60)		Controls(*n* = 60)		LN(*n* = 20)		APS(*n* = 20)		Skin and joint*n* = 20)	
Variables	*B*	*P*	*B*	*P*	*B*	*P*	*B*	*P*	*B*	*P*
Background variables										
** **Age	0.89	0.73	–0.07	0.67	0.55	0.13	–0.54	0.33	0.17	0.59
** **Female gender	8.20	0.33	3.62	0.51	–3.57	0.78	9.21	0.55	–6.05	0.72
** **Duration of SLE	**0.63**	**0.04**	0	0	0.43	0.36	0.77	0.15	0.70	0.23
** **SDI score	**9.74**	**<0.001**	0	0	**9.20**	**0.02**	**10.30**	**0.02**	–6.20	0.42
** **SLEDAI-2K	2.71	**0.05**	0	0	–0.27	0.91	2.60	0.35	**4.53**	**0.005**
Traditional risk factors and laboratory data										
** **BMI (kg/m^2^)	0.37	0.59	–0.28	0.61	0.92	0.42	–0.37	0.83	–0.71	0.33
** **Ever smoker (former or current)	0.83	0.90	0	0	2.35	0.80	–7.21	0.64	6.13	0.47
** **Systolic blood pressure	0.23	0.21	0.19	0.17	**0.48**	**0.02**	0.02	0.95	0.15	0.57
** **Diastolic blood pressure	0.27	0.32	0.16	0.48	0.40	0.20	0.13	0.85	0.20	0.62
** **Raynaud’s phenomenon	**12.37**	**0.05**	–7.85	0.12	3.21	0.80	17.76	0.19	6.90	0.37
** **eGFR	–0.07	0.69	0	0	0.12	0.61	–0.45	0.35	0.01	0.95
** **Total cholesterol	1.94	0.50	–0.28	0.86	2.01	0.61	–3.17	0.69	4.08	0.21
** **High-density lipoprotein (HDL)	6.73	0.32	1.37	0.76	7.33	0.46	–4.31	0.74	19.15	0.06
** **Low-density lipoprotein (LDL)	–0.81	0.81	–0.40	0.83	–1.71	0.70	–3.30	0.74	3.57	0.38
** **Triglycerides (TG)	4.40	0.27	–1.50	0.63	11.45	0.06	0.70	0.92	0.70	0.94
** **hsCRP	**2.16**	**0.03**	0.19	0.71	1.66	0.59	**4.10**	**0.03**	–0.51	0.66
Medical treatment, ongoing										
** **Antimalarial agents	–1.73	0.86	N/A	N/A	NE	NE	–0.33	0.98	12.57	0.30
** **Glucocorticoid therapy	4.98	0.38	N/A	N/A	12.73	0.08	3.45	0.80	3.50	0.63
** **Warfarin therapy	**22.0**	**0.002**	N/A	N/A	9.15	0.60	18.90	0.14	NE	NE
** **Antiplatelet therapy	–6.60	0.37	N/A	N/A	4.67	0.59	–26.0	0.06	NE	NE
** **Statin therapy	7.50	0.47	N/A	N/A	–18.62	0.27	5.43	0.74	NE	NE
** **DMARD therapy	–1.76	0.76	N/A	N/A	–4.75	0.53	–6.01	0.65	1.69	0.82

APS: antiphospholipid syndrome; BMI: body mass index; CRP: C-reactive protein; DMARDs: disease modifying anti-rheumatic drugs; LN: lupus nephritis; N/A: not applicable; NE: Not estimated; SDI: SLICC/ACR damage index; SLE: systemic lupus erythematosus. Note: Statistically significant associations shown in bold.

### Correlation of CCA IMT in each subgroup and studied variables

Correlations between background variables and laboratory measurements and mean CCA IMT are shown in Table 3. Statistically significant correlations were mainly observed with SDI, tobacco smoking, triglycerides and use of antimalarial agents. In the control group, age, blood pressure (both systolic and diastolic), cholesterol and LDL levels were correlated with CCA IMT. The analysis was stratified, and the included variables tested in relation to mean CCA IMT right and left sides, as shown in Table 4. A significant association was observed with blood pressure and age in the controls and with the SLE duration, acquired organ damage (SDI score) and lipid levels (TG and total cholesterol) in SLE cases (*n* = 60).

**Table 3.** Mean CCA IMT (bilateral) related to background variables, traditional risk factors, laboratory tests and pharmacotherapy in univariate regression model of SLE cases with subgroups compared to healthy controls.

	All SLE(*n* = 60)		Controls(*n* = 60)		LN(*n* = 20)		APS(*n* = 20)		Skin and joint(*n* = 20)	
Variables	*B*	*P*	*B*	*P*	*B*	*P*	*B*	*P*	*B*	*P*
Background variables											
** **Age	–0.00	0.62	**0.01**	**<0.001**	–0.00	0.33	–0.00	0.72	–8.90	0.95
** **Female gender	0.06	0.15	–0.00	0.99	0.07	0.19	0.06	0.44	–0.08	0.29
** **Duration of SLE	0.00	0.06	0	0	0.00	0.62	0.00	0.34	0.00	0.48
** **SDI score	**0.03**	**0.01**	0	0	0.00	0.91	0.04	0.14	0.03	0.39
** **SLEDAI-2K	0.00	0.76	0	0	–0.02	0.07	–0.00	0.88	0.01	0.46
Traditional risk factors and laboratory data											
** **BMI (kg/m^2^)	0.00	0.79	**0.01**	**0.05**	0.00	0.58	0.01	0.60	–0.01	0.63
** **Ever smoker (former or current)	**0.09**	**0.01**	0	0	**0.10**	**0.02**	–9.40	0.96	0.02	0.66
** **Systolic blood pressure	2.84	0.97	**0.00**	**<0.001**	0.00	0.74	0.02	0.95	–3.17	1.00
** **Diastolic blood pressure	–0.00	0.59	**0.00**	**0.02**	0.00	0.86	–0.00	0.44	0.00	0.87
** **Raynaud’s phenomenon	0.05	0.15	–0.03	0.33	0.01	0.84	**0.15**	**0.03**	–0.04	0.28
** **Estimated glomerular filtration rate	0.00	0.45	0	0	**0.00**	**0.04**	–0.00	0.54	0.00	0.45
** **Total cholesterol	0.02	0.22	**0.05**	**<0.001**	0.01	0.64	0.07	0.10	–0.00	0.83
** **High-density lipoprotein (HDL)	0.03	0.30	0.06	0.09	0.02	0.74	–0.09	0.17	–0.01	0.78
** **Low-density lipoprotein (LDL)	0.01	0.50	**0.05**	**<0.001**	0.01	0.72	0.07	0.16	–0.00	0.84
** **Triglycerides (TG)	**0.06**	**<0.001**	0.01	0.65	0.01	0.68	**0.09**	**0.01**	0.01	0.89
** **High-sensitivity CRP	0.01	0.34	0.00	0.57	0.00	0.96	0.01	0.40	–0.00	0.90
Medical treatment, ongoing											
** **Antimalarial agents	**–0.12**	**0.01**	0	0	NE	NE	**–0.19**	**0.02**	0.03	0.66
** **Glucocorticoid therapy	**–**0.02	0.51	0	0	0.05	0.15	–0.06	0.36	–0.03	0.39
** **Warfarin therapy	**0.07**	**0.05**	0	0	0.05	0.50	0.05	0.49	NE	NE
** **Antiplatelet therapy	–0.05	0.16	0	0	–0.01	0.84	–0.14	0.06	NE	NE
** **Statin therapy	–0.01	0.88	0	0	**–0.16**	**0.04**	–0.00	0.96	NE	NE
** **DMARD therapy, n (%)	–1.76	0.76	0	0	0.01	0.88	**0.14**	**0.03**	**0.10**	**0.001**

APS: antiphospholipid syndrome; CRP: C-reactive protein; BMI: body mass index; DMARDs: disease modifying anti-rheumatic drugs; LN: lupus nephritis; N/A: not applicable; NE: Not estimated; SDI: SLICC/ACR damage index; SLE: systemic lupus erythematosus.

Note: Statistically significant associations shown in bold.

**Table 4.** Mean CCA IMT in right and left side in all SLE group and controls related to included variables.

Variables	Controls: CCA IMT (n = 60)				All SLE: CCA IMT (n = 60)			
	Right CCA		Left CCA		Right CCA		Left CCA	
	*B*	*P*	*B*	*P*	*B*	*P*	*B*	*P*
** **Age	**7.95**	**0.014**	2.78	0.37	0.01	0.91	0.14	0.26
** **Female gender	–110.64	0.36	72.60	0.51	–2.83	0.48	–5.14	0.20
Duration of SLE	0	0	0	0	0.15	0.31	**0.35**	**0.02**
SDI score	0	0	0	0	0.35	0.77	**3.07**	**0.01**
SLEDAI-2K	0	0	0	0	0.30	0.65	0.04	0.95
Traditional risk factors and laboratory data								
BMI (kg/m^2^)	11.6	0.30	16.42	0.12	–0.16	0.64	–0.30	0.37
Ever smoker (former or current)	0	0	0	0	–1.05	0.74	–1.10	0.74
Systolic blood pressure	**8.60**	**0.002**	3.03	0.26	–0.03	0.76	0.10	0.25
Diastolic blood pressure	**11.22**	**0.014**	2.75	0.53	–0.01	0.95	0.19	0.16
Raynaud	–105.00	0.32	–149.94	0.11	–0.87	0.78	0.58	0.85
Estimated glomerular filtration rate	0	0	0	0	–0.04	0.68	0.10	0.25
Total cholesterol	21.21	0.53	34.38	0.27	1.37	0.32	**3.07**	**0.03**
High-density lipoprotein (HDL)	138.84	0.14	5.24	0.95	0.02	1.00	5.83	0.07
Low-density lipoprotein (LDL)	3.10	0.94	51.04	0.17	0.06	0.52	1.18	0.48
Triglycerides (TG)	19.14	0.77	–17.60	0.77	3.10	0.09	**4.81**	**0.01**
High-sensitivity CRP	–9.26	0.40	–6.30	0.53	–0.10	0.84	0.26	0.59
Medical treatment, ongoing								
Antimalarial agents	0	0	0	0	–5.98	0.18	–2.56	0.58
Glucocorticoid therapy	0	0	0	0	–3.80	0.16	–0.10	0.70
Warfarin therapy	0	0	0	0	–1.55	0.66	–1.36	0.70
Antiplatelet therapy	0	0	0	0	–5.34	0.12	2.76	0.44
Statin therapy	0	0	0	0	–4.42	0.37	–4.80	0.34
DMARD therapy	0	0	0	0	1.60	0.55	3.75	0.17

APS: Antiphospholipid syndrome; CRP: C-reactive protein; BMI: body mass index; DMARDs: disease modifying anti-rheumatic drugs; LN: lupus nephritis; SDI: SLICC/ACR damage index; SLE: systemic lupus erythematosus.

Notes: Statistically significant associations shown in bold.

No obvious differences in bilateral mean CCA IMT between SLE patients and controls, as well as between the SLE subgroups and controls were seen (Figure 1(b)).

### Multivariable regression analysis for pOPN and CCA IMT

Next, variables showing significant associations with pOPN or CCA IMT in the univariate regression model were analysed in a multivariable regression model. As shown in Table 5, only the global SDI score remained significantly associated with pOPN levels, whereas smoking (ever) and triglycerides were significantly associated with CCA IMT.

**Table 5.** Multivariable regression analysis for significant associations between evaluated variables and plasma osteopontin (pOPN), as well as CCA IMT in the SLE patients (*n* = 60).

	Univariate analysis		Multivariable analysis	
Risk factors	*B*	*P value*	*B*	*P value*
pOPN
SLE duration	0.63	**0.04**	–0.24	0.50
SDI score	9.74	**0.00**	7.02	**0.04**
hsCRP	2.16	**0.03**	1.55	0.10
Raynaud	12.37	**0.05**	8.12	0.15
Warfarin	22.0	**0.002**	13.27	0.08
CCA IMT				
SDI score	0.03	**0.01**	0.010	0.44
Tobacco smoking (ever)	0.09	**0.01**	0.06	**0.05**
Triglycerides	0.06	**0.00**	0.04	**0.04**
Antimalarials	–0.12	**0.01**	–0.07	0.10
Warfarin	0.07	**0.05**	0.01	0.81

hsCRP: high-sensitivity C-reactive protein; SDI: SLICC/ACR damage index; SLE: systemic lupus erythematosus.

Note: Statistically significant associations shown in bold.

### Correlation between pOPN and CCA IMT

Finally, the potential role of pOPN as a surrogate marker of CCA IMT was evaluated. No significant correlations between pOPN levels and CCA IMT were found in any of the included groups (Table 6).

**Table 6.** Correlations between plasma osteopontin (pOPN) and CCA IMT in the studied groups and subgroups in a univariate regression model.

pOPN levels	CCA IMT	
	*B*	*P*
Healthy controls	0.00	0.72
All SLE cases	0.00	0.38
SLE with lupus nephritis	0.00	0.91
SLE with antiphospholipid syndrome	0.00	0.85
SLE with skin & joint involvement	0.00	0.79

## Discussion

The aims of the present study were to evaluate the potential of pOPN as a surrogate marker mirroring the CCA IMT, assessed by using HFUS, to investigate subclinical atherosclerosis in well-characterized SLE subjects and healthy controls. Entirely in line with previous reports,^18,31,34,35^ OPN was significantly higher in patients with SLE than in healthy controls, but its association with subclinical atherosclerosis was poor, which is not consistent with the findings reported by Carbone et al.^21^

The discrepant findings may be explained by several factors, such as the higher frequency we utilized in our HFUS investigations, the fact that OPN was measured in plasma (instead of serum) and the presence of males (13%) in our study populations. Sex bias could be relevant since male SLE patients often have worse prognosis than women.^36^ Furthermore, our data were adjusted for SDI, disease phenotypes and ongoing pharmacotherapy, including treatments with important effects on both atherogenesis and vessels changes, e.g. corticosteroids and DMARDs, especially antimalarials. We believe it is critical to contemplate damage accrual (assessed by the SDI) in such a study, as it has been recognized that damage predicts further damage in SLE.^37,38^

We did not detect any considerable differences in pOPN levels between the SLE phenotypic subgroups. Previous reports on larger study populations have shown that LN is associated with higher OPN levels compared to other disease phenotypes, but the impact of SLE disease activity cannot be excluded.^31,34,35^ Herein, most patients had clinically quiescent disease and the SLEDAI-2K did not differ significantly between the phenotypic subgroups.

In the subgroup with isolated skin and joint involvement, pOPN was significantly correlated with some SLE-related factors, such as disease duration, SDI and disease activity. These observations are mainly in agreement with previous findings, where elevated levels of OPN preceded increased cumulative SLE disease activity and organ damage.^31^

OPN has been implicated as a mediator of Th17 regulation via type I IFN receptor signalling, in macrophage activity at sites of tissue repair and in bone homoeostasis.^18,39,40^ OPN contributes to macrophage chemotaxis, activation, survival and pro-inflammatory M1 polarization. In addition, OPN promotes neutrophil recruitment and activation.^41^ Regarding SLE and atherosclerosis, OPN plays a role through regulation of type I IFN response, which is considered as part of pathophysiology in both conditions.^42,43^

Warfarin therapy showed a prominent positive association with pOPN levels. Warfarin is a vitamin K antagonist and may give vitamin K deficiency causing vascular calcification through inhibition of calcification inhibitors, including gamma-carboxyglutamic acid, Gla protein, fetuin and OPN.^44,45^ CCA IMT had significant correlations in our study with traditional factors such as age, smoking, blood pressure and lipid (TG, LDL) levels in controls, SLE patients and subgroups. SDI of all SLE cases were also slightly correlated with CCA IMT. A negative low association was observed between CCA IMT and treatment with statins, as well as with antimalarial agents.

Antimalarials have a known cholesterol-lowering effect, especially in SLE patients with concomitant corticosteroid treatment.^46,47^ In the SLE subgroups with APS and skin and joints involvement, a minor positive association was observed between DMARD therapy and CCA IMT. Similarly to this finding, long-term use of DMARDs in patients with rheumatoid arthritis was reported to develop incident hyperlipidaemia.^48^ Following a multivariable analysis of the studied variables or risk factors against both elevated pOPN or increased CCA IMT, only a few factors still had significant associations (as shown in Table 5).

We could not find any associations between pOPN and CCA IMT in the SLE group, not even after stratifying into different disease phenotypes. Despite the multiple possible mechanistic roles of OPN in atherogenesis in SLE patients,^17,19,31^ pOPN levels did not mirror the wall thickness of CCA. This may be due to the younger age of the patients in our study,^22^ differences in SLE duration, or possibly obscured by warfarin treatment in approximately one fifth of the patients. Yet, although warfarin can cause arterial wall calcification, the vascular wall changes in atherosclerosis are different from vascular calcification. Arteriosclerosis makes the artery wall thicker as a result of invasion and accumulation of white blood cells (foam cells) and the proliferation of intimal smooth muscle cells, creating fibro-fatty plaques, while vascular calcification is present mainly in the smooth muscle layer of arteries leading to impairment of the vascular tone and consequent arterial stiffness.^49,50^

The limitations in the present study are mainly due to the relatively small number of included subjects. The low age of the selected patients may have affected the limited vascular changes. The well-characterized SLE patients’ group and the study design with stratification of data according to different clinical phenotypes and well-matched controls represent major strengths of our investigation. Moreover, the accuracy of the HFUS examination is high with two repeated measurements in each side in every subject performed by the same examiner.

To summarize, we evaluated the vessel wall appearance of CCA using sensitive HFUS technique in well-characterized SLE subjects and matched controls. Although pOPN levels were significantly increased among the patients and associated with both traditional and SLE-related risk factors, the pOPN concentrations did not correlate significantly with CCA IMT findings. We propose that the pOPN levels should not be used as a surrogate marker of atherosclerosis in patients with SLE, regardless of disease phenotype.

## Supplemental Material

sj-pdf-1-lup-10.1177_09612033211013898 - Supplemental material for Plasma osteopontin versus intima media thickness of the common carotid arteries in well-characterised patients with systemic lupus erythematosus Click here for additional data file. Supplemental material, sj-pdf-1-lup-10.1177_09612033211013898 for Plasma osteopontin versus intima media thickness of the common carotid arteries in well-characterised patients with systemic lupus erythematosus by Lina Wirestam, Muna Saleh, Christina Svensson, Michele Compagno, Helene Zachrisson, Jonas Wetterö and Christopher Sjöwall in Lupus
