# Supplementary figures and images for: Plasma osteopontin versus intima media thickness of the common carotid arteries in well-characterised patients with systemic lupus erythematosus
Source: Lupus. 2021 May 6;30(8):1244–53. doi: 10.1177/09612033211013898 (PMC8209759; doi:10.1177/09612033211013898)

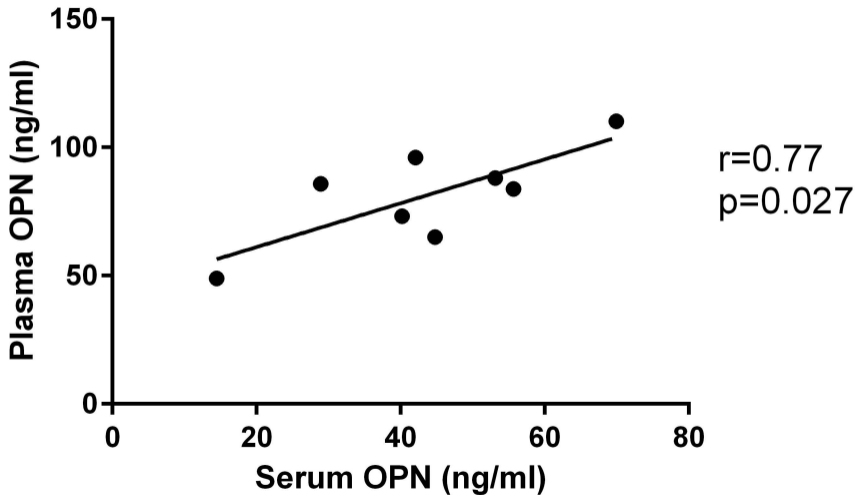

Supplement: sj-pdf-1-lup-10.1177_09612033211013898 - Supplemental material for Plasma osteopontin versus intima media thickness of the common carotid arteries in well-characterised patients with systemic lupus erythematosus [file sj-pdf-1-lup-10.1177_09612033211013898.pdf]
